# A Novel Methodology to Synthesize Highly Conductive Anion Exchange Membranes

**DOI:** 10.1038/srep13417

**Published:** 2015-08-27

**Authors:** Yubin He, Jiefeng Pan, Liang Wu, Yuan Zhu, Xiaolin Ge, Jin Ran, ZhengJin Yang, Tongwen Xu

**Affiliations:** 1 CAS Key Laboratory of Soft Matter Chemistry, Collaborative Innovation Center of Chemistry for Energy Materials, School of Chemistry and Material Science, University of Science and Technology of China, Hefei 230026, P.R

## Abstract

Alkaline polyelectrolyte fuel cell now receives growing attention as a promising candidate to serve as the next generation energy-generating device by enabling the use of non-precious metal catalysts (silver, cobalt, nickel *et al.*). However, the development and application of alkaline polyelectrolyte fuel cell is still blocked by the poor hydroxide conductivity of anion exchange membranes. In order to solve this problem, we demonstrate a methodology for the preparation of highly OH^−^ conductive anion exchange polyelectrolytes with good alkaline tolerance and excellent dimensional stability. Polymer backbones were grafted with flexible aliphatic chains containing two or three quaternized ammonium groups. The highly flexible and hydrophilic multi-functionalized side chains prefer to aggregate together to facilitate the formation of well-defined hydrophilic-hydrophobic microphase separation, which is crucial for the superior OH^−^ conductivity of 69 mS/cm at room temperature. Besides, the as-prepared AEMs also exhibit excellent alkaline tolerance as well as improved dimensional stability due to their carefully designed polymer architecture, which provide new directions to pursue high performance AEMs and are promising to serve as a candidate for fuel cell technology.

Fuel cell technology is recognized as one of the most promising candidate to cope with the impending energy crisis. Compared to proton exchange membrane fuel cell (PEMFC), alkaline polyelectrolyte fuel cell (APEFC) processes significant advantages by employing non-precious catalyst (silver, cobalt, nickel *et al.*) and improving the kinetic of oxygen reduction^1^. However, the development of APEFC is still facing a severe challenge due to low hydroxide conductivity caused by the inherently lower conductive ratio of OH^−^ relative to H^+^. On the other hand, compared with Nafion®, the state of art proton exchange membrane which demonstrates excellent proton conductivity attributing to its particular comb-shaped polymer architecture and the strong acidity of sulfonic acid groups, poor micro phase separation ability and weak basicity of quaternary ammonium hydroxide are also considered as the key problems for improving the OH^−^ conductivity of anion exchange membranes (AEMs).

To date, numerous strategies have been applied to improve the hydroxide conductivity of AEMs which can be mainly classified into two categories: synthesis of new anion conductive groups and design of polymer architecture^2^,^3^ for tuning of ordered micro-phase segregations. Up to now, various ion conductive groups^4^,^5^,^6^,^7^ have been investigated as new side groups of AEMs instead of the conventional quaternary ammonium groups (QA). However in order to improve the basicity of ionic moieties of AEMs thus can lead to better dissociation ability based on an overall consideration of hydroxide conductivity, alkaline stability and manufacture cost, quaternary ammonium moieties are still the state of art hydroxide conducting groups for AEMs.

Ordinarily, QA groups based anion exchange membranes are prepared either by chloromethylation or bromomethylation followed by quaternization, which leads to AEMs with quaternary ammonium groups closely attached to the polymer backbones. This kind of anion exchange membranes usually show low hydroxide conductivity ascribing to their less ordered self-assembly morphologies as previously reported^8^. However, this problem can be partially solved by looking into acidic analogues. As we know, Nafion® processes outstanding microphase separation ability because of its highly flexible side chain. Inspired by this, various side chain type proton exchange polyelectrolytes have been synthesized and proved to process superior performance in proton conductivity over their analogue with sulfonic acid groups randomly located along the aromatic chains^9^,^10^,^11^. Similarly, the attachment of a long flexible side chains terminated by anion conducting groups is also considered to facilitate the formation of microphase separation of AEMs. And as an experiential verification of this proposal, side chain type anion exchange membranes were successfully developed in our lab via poly-condensation of pre-quaternized monomers for morphology tuning purpose, and as-expected good microphase separation and high OH^−^ conductivity were observed^12^.

To further improve the phase separation ability of AEMs, incorporation of longer aliphatic chains with more flexibility between QA groups and polymer backbones may be the simplest way, but inferior hydrophilicity of QA moieties compared with sulfonic acid groups seems impossible to be overcome. However, hydrophilicity of one polymer segment can be effectively improved by densely grafting of QA groups^13^. Because multi-functionalized segments with densely located quaternized ammonium groups are more hydrophilic than mono-functionalized ones and prefer to aggregate together to facilitate the formation of hydrophilic-hydrophobic microphase separation, thus higher hydroxide conductivity of this types of AEMs were also observed. However, it should be noted that grafting of more positively charged QA groups on one phenyl ring may risk easier attack of hydroxide ion to the α-carbon of nitrogen because of electro-withdrawing effect of QA groups, thus lead to poor alkaline stability. Besides, most of the multi-QA grafted segments were constructed from aromatic rings, which lack enough flexibility to form better defined phase separation. Hence, we propose that both hydrophilicity and flexibility of functionalized segments are crucial to obtain AEMs with well-ordered microphase segregation. Designing of particular polymer architecture with highly flexible side chains densely functionalized by QA groups^14^ should be the best method to demonstrate this proposal, which is a combination of side chains architecture and densely functionalized strategy.

## Results and Discussion

### Synthesis

**Figure 1:**
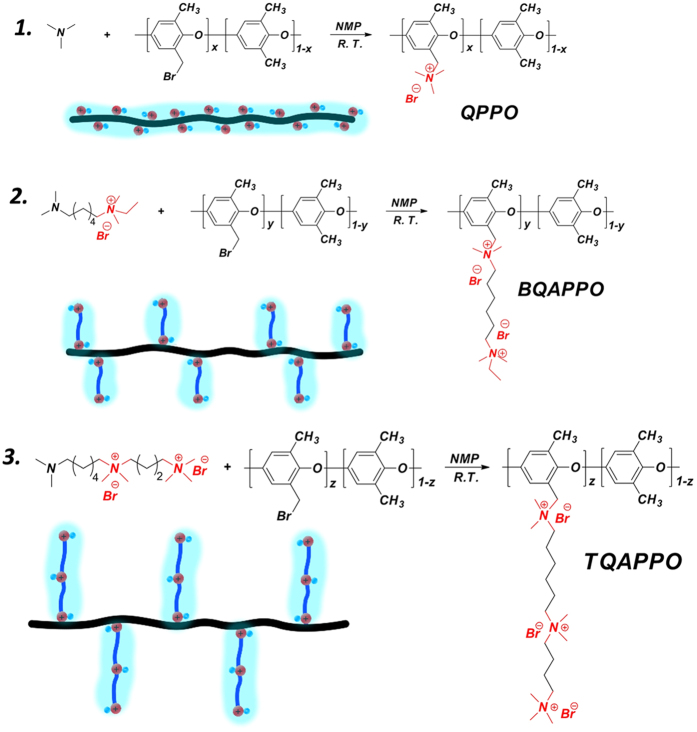
Synthetic procedures of QPPO, BQAPPO and TQAPPO.

As experiential verification of this proposal, 6-(dimethylamino)-N-ethyl-N,N-dimethylhexan-1-aminium bromide (DMAQA) with one tertiary amine and one QA group was synthesized via two steps of Menshutkin reactions (Figure S1). Afterwards, DMAQA was reacted with brominated poly(2,6-dimethyl-1,4-phenylene oxide) (BPPO) at room temperature to yield polyelectrolytes functionalized by a flexible side chain which was incorporated by two cations. (BQAPPO, Fig. 1). And in order to further enhance the microphase separation ability of AEMs, polyelectrolytes grafted with super flexible side chains which were incorporated by three QA groups were similarly synthesized employing three steps of Menshutkin reactions (TQAPPO, Fig. 1). Key properties of the synthesized AEMs were listed in Table S1. It should be noted that every steps involved process in mild conditions and all start from commercially available inexpensive materials, which is possible to be scaled up and applied in the field of fuel cell technology.

**Figure 2:**
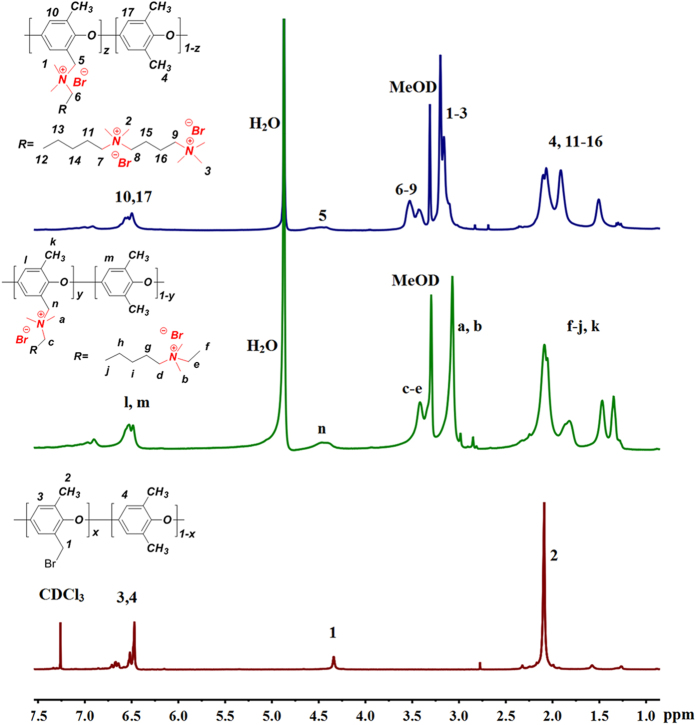
^1^H NMR spectra of BPPO, BQAPPO and TQAPPO.

In order to demonstrate the as designed polymer structure of synthesized polyelectrolytes, ^1^H NMR spectrums of BPPO, BQAPPO and TQAPPO were depicted in Fig. 2 for comparison. New signals of N–CH_3_ groups appear at around 2.9–3.3 ppm for both BQAPPO and TQAPPO, which was in accordance with that of DMAQA (Figure S2) or DMABQA (Figure S3). For BPPO, there was only one peak at low chemical shift region of 2.0–2.15 ppm, new peaks of this region observed for the synthesized polyelectrolytes were assigned to the –C–CH_2_–C– groups in the aliphatic chain of functional moieties. Accordant with that of DMAQA and DMABQA, new peaks appear at 3.35–3.45 ppm were assigned to the N-connected –CH_2_– groups. Compared to BQAPPO, there are two peaks at this region for TQAPPO, which implies there are two types of N-connected –CH_2_– groups, corresponding to the diversity structure feature between BQAPPO and TQAPPO, which is crucial for the superior performance of tri-QA type anion exchange membranes.

### Hydroxide Conductivity

**Figure 3:**
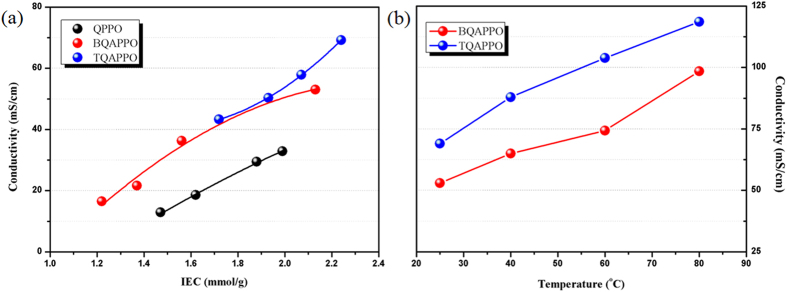
(a) IEC dependent hydroxide conductivity of QPPO, BQAPPO and TQAPPO at 25 °C; (b) Hydroxide conductivities of BQAPPO (IEC = 2.13 mmol/g) and TQAPPO (IEC = 2.24 mmol/g) membranes as a function of temperature.

The original intention of this work is to improve the hydroxide conductivity of AEMs by enhancing its hydrophilic-hydrophobic phase segregation ability. Since QA groups bear instinct disadvantages of low basicity and incapability to form connected hydrogen bonds network, polymer architecture of AEMs should be carefully designed to obtain comparable conductivity as proton exchange membranes. In order to demonstrate the superiority of this type of AEMs with flexible and densely functionalized side chains, quaternized poly(2,6-dimethyl-1,4-phenylene oxide) (QPPO) was prepared via homogeneous reaction between BPPO and trimethylamine. As depicted in Fig. 3a, BQAPPO shows obviously higher OH- conductivity (53 mS/cm) than QPPO (33 mS/cm) at similar IEC values (~2.1 mmol/g) attributing to high mobility of QA groups resulting from the higher flexibility of side chains. Moreover, since its side chain was densely incorporated by two QA groups which can effectively enhance the hydrophilic-hydrophobic difference of side chains and polymer backbones to result better self-assembly morphologies, BQAPPO also exhibits superior performance than previously reported side chain type AEMs^12^,^15^. By increasing the length and number of incorporated cations of side chain, dramatically enhanced hydroxide conductivity was observed for TQAPPO (69 mS/cm) at room temperature, which is much higher than the values of AEMs with densely functionalized segments^16^. These results clearly verified that mobility and hydrophilicity of functional moieties both play crucial roles to achieve high performance AEMs, by promoting the aggregation of hydrophilic segment to result well defined phase segregation and inter-connected ion conducting channels. Besides, high density of positively charged QA groups on the side chain can also lead to higher cation concentration of hydrophilic domains, which will efficiently promote the hoping of hydroxide ions. Considering that fuel cells may operate at high temperatures in order to improve kinetic of reactions on the electrodes, hydroxide conductivity at different temperatures ranging from 30 °C to 80 °C was measured (Fig. 3b). Because of both enhanced mobility of OH^−^ and QA groups, outstanding conductivity of 98 mS/cm and 119 mS/cm was observed for BQAPPO and TQAPPO at 80 °C separately.

### Morphology

**Figure 4:**
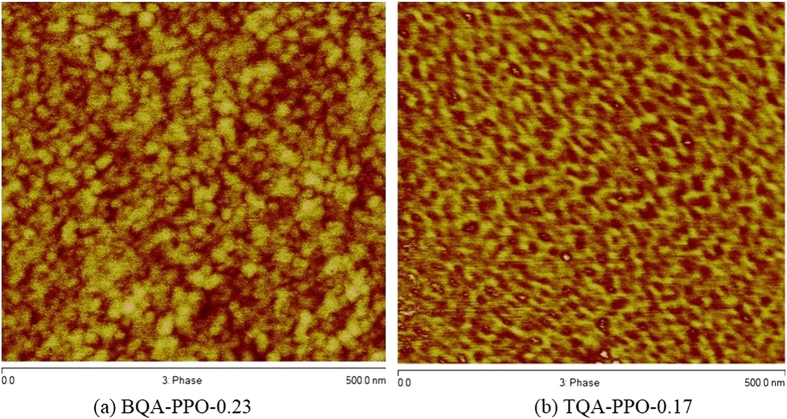
AFM tapping phase images of BQAPPO (a) and TQAPPO (b).

In order to further reveal the architecture-morphology-properties relationship of AEMs, tapping mode atomic force microscopy (AFM) was employed to investigated the self-assembly morphologies of the synthesized membranes. It is well-known that the hydrophilic-hydrophobic difference between functionalized side chains and polymer backbones is crucial for the formation of well-defined micro-phase segregation and interconnect hydroxide conducting channels. With increased flexibility and hydrophilicity of side chains, obvious phase-separation morphologies of BQAPPO and TQAPPO (Fig. 4) were observed at similar IEC values. TQAPPO displays best phase-separation ability and nanoscale ion conducting clusters that could be clearly observed throughout the field of views. This further interprets the outstanding hydroxide conductivity of 69 mS/cm and clearly confirms our original proposal that both hydrophilicity and flexibility of ionic side chains play crucial parts in the fabrication high performance AEMs.

### Alkaline stability

**Figure 5:**
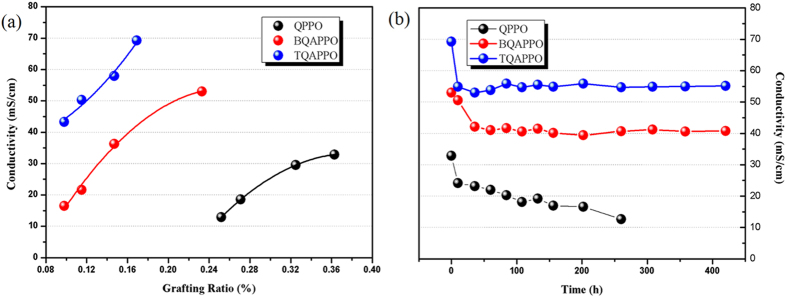
(a) Grafting ratio dependent hydroxide conductivity of QPPO, BQAPPO and TQAPPO at 25 °C; (b) Alkaline stability of BQAPPO and TQAPPO measured in 1 mol/L KOH at 60 °C.

Another crucial property of AEMs is alkaline stability which is correlated to the lifetime of membrane in fuel cell operation conditions. Pristine BPPO is rather tolerant to alkaline environment, however, after quantization, the polymer main chain degenerates quickly when exposed to concentrated KOH solution^17^. This implies the incorporation of cationic QA groups will inevitably alter the stability of BPPO backbones. So, in order to disentangle the stability-conductivity dilemma, high IEC values and OH^−^ conductivity must be simultaneously obtained at low grafting ratio^18^. By verifying the bromination degree of BPPO, grafting ratio of the synthesized polyelectrolytes was easily controlled, and as depicted in Fig. 5a, IEC values more than 2.0 mmol/g only acquire grafting ratio of 23% and 17% for BQAPPO and TQAPPO respectively. Moreover, to obtain similar OH^−^ conductivity of 33 mS/cm as QPPO, the grafting ratio of TQAPPO (10%) and BQAPPO (15%) is about several times lower than that of QPPO (36%). This may be one of the reasons for the superior chemical stability of the synthesized AEMs over QPPO AEMs (Fig. 5b). Further, degeneration of polymer backbones of the synthesized AEMs was investigated by measuring the change of mechanical properties before and after alkaline treatment. BQAPPO and TQAPPO AEMs maintained 68% and 52% of its initial tensile strength after soaking in 1 M KOH aqueous solution at 60 °C for 15 days. However, QPPO AEM became so brittle after 10 days of alkaline treatment thus its mechanical properties cannot be measured (Table S2). This result demonstrated that our strategy could effectively enhance the stability of polymer backbone.

Besides the degeneration of polymer backbones, β-hydrogen Hofmann elimination and direct nucleophilic substitution at α-carbon are also considered as well-known degeneration pathways of quaternary ammonium groups in basic environment^19^. It should be noted that QA group directly attached to benzyl is not as stable as the aliphatic chain connected QA moieties because of the electro-withdrawing effect of phenyl rings. However, by adding a long aliphatic chain attaching to nitrogen atom can profoundly improve the chemical stability of benzyl connected QA groups^20^. Attributing to the steric hindrance effect of side chains, as synthesized polyelectrolytes display better alkaline stability than QPPO, due to the decreased chance of attack to benzyl connected QA group by hydroxide ions. Furthermore, length of the aliphatic spacer between two cations also has profound impact on chemical stability of AEMs^21^. Aliphatic spacer with less than three carbon atoms is proved to be unstable because of increased acidity of β-hydrogen caused by the electro-withdrawing effect of positively charged QA groups. So in order to obtain alkaline tolerant AEMs, two QA groups must be separated by more than four carbon atoms. And as a result of above mentioned advantages, both BQAPPO and TQAPPO exhibit excellent alkaline stability of remaining more than 77% initial conductivity after soaking in 1 M KOH at 60 °C for more than 400 h.

### Water uptake

**Figure 6:**
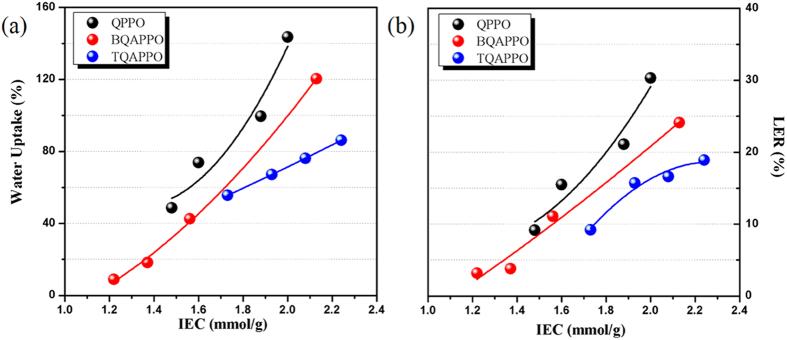
(a) IEC dependent water uptake of QPPO, BQAPPO and TQAPPO at 25 °C (OH^−^ form); (b) IEC dependent liner expansion ratio of QPPO, BQAPPO and TQAPPO at 25 °C (OH^−^ form).

As we know, polymer backbones of main chain type AEMs are densely functionalized by QA groups in order to obtain high IEC values. Because of the hydrophilicity of QA groups, polymer main chains are in fact warped by water molecular, which leads to poor micro phase separation and high water uptake. By reducing the graft ratio on the main chains and increasing the charge density of side chains, particular polymer architecture composed of hydrophobic main chains and hydrophilic side chains was obtained, resulting better defined microphase separation which can restrict the excess absorption of water. As depicted in Fig. 6a, lower water uptake was observed with decreasing grafting ratio at similar IEC values because of more hydrophobic segments on the mainchain. Furthermore, high charge density of side chains can also greatly enhance the intermolecular interaction of polymer chain, thus lead to lower expansion ratio (LER) of multi-cations functionalized AEMs than QPPO at similar IEC values (Fig. 6b, TQAPPO: 19%; BQAPPO: 24%; QPPO: 30%).

## Conclusions

In summary, we demonstrated a facile but efficient methodology for the synthesis of highly OH^−^ conductive AEMs. By grafting flexible side chain containing two or three QA moieties onto the polymer backbones, OH^−^ conductivity of the synthesized multi-cations functionalized AEMs can be remarkably improved to as high as 69 mS/cm at room temperature, even comparable to that of Nafion® though theoretical migration of H^+^ is much higher than that of OH^−^. Despite the outstanding performance in OH^−^ conductivity, this methodology can also remarkably decrease the swell ratio of the AEMs as well as improving the chemical stability due to its carefully designed polymer architecture.

## Methods

### Synthesis of multi-cations functionalized AEMs (BQAPPO and TQAPPO)

To a stirred solution of 1 g BPPO in 10 mL NMP was added 1.2 equiv of DMAQA or DMABQA followed by stirring at room temperature for 24 hours. Afterwards, it was poured into excess ether and synthesized polyelectrolyte was collected by filtration and washed with ether for several times. After dried at 60 °C for 24 hours, the polymer (1 g) was dissolve in NMP (15 mL) then casted onto glass plate and heated at 60 °C to form transparent membrane. Key properties of the synthesized AEMs were listed in Table S1.

## Additional Information

**How to cite this article**: He, Y. *et al.* A Novel Methodology to Synthesize Highly Conductive Anion Exchange Membranes. *Sci. Rep.* **5**, 13417; doi: 10.1038/srep13417 (2015).

## Supplementary Material

Supplementary Information
