# Supplementary material for: A Novel Methodology to Synthesize Highly Conductive Anion Exchange Membranes
Source: Sci Rep. 2015 Aug 27;5:13417. doi: 10.1038/srep13417 (PMC4550832; doi:10.1038/srep13417)
Supplement: Supplementary Information [file srep13417-s1.doc]

**A Novel Methodology to Synthesize Highly Conductive Anion Exchange Membranes**

Yubin He,† Jiefeng Pan,† Liang Wu, Yuan Zhu, Xiaolin Ge, Jin Ran, ZhengJin Yang, and Tongwen Xu^*^

CAS Key Laboratory of Soft Matter Chemistry, Collaborative Innovation Center of Chemistry for Energy Materials, School of Chemistry and Material Science, University of Science and Technology of China, Hefei 230026, P.R. Tel.: +86-551-6360-1587.E-mail:twxu@ustc.edu.cn.

† These authors contribute equally.

^*^Corresponding author: Tel.: +86-551-6360-1587.E-mail:twxu@ustc.edu.cn.

**Materials:**

Poly(2,6-dimethyl-1,4-phenylene oxide) (PPO) with an intrinsic viscosity of 0.57 dl/g in chloroform at 25 ^o^C was obtained from Tianwei Membrane Company (Shandong, P.R. China). N-bromosuccinimide (NBS), 2,2'-azobis(2-methylpropionitrile) (AIBN), N,N,N',N'-Tetramethyl-1,6-hexanediamine (TMHDA), bromoethane and 1,4-dibromobutane was purchased from Energy Chemical Co. Ltd.(Shanghai, P.R. China) and used as received. N-methyl-2-pyrrolidolone (NMP, AR), tetrahydrofuran (THF), CH_3_CN, acetone, ether, chlorobenzene, ethanol, chloroform, trimethylamine aqueous solution (33 %), Hydrochloric acid (HCl) aqueous solution (37% AR), sodium chloride (AR), sodium hydroxide (AR) and sodium sulfate (Na_2_SO_4_, AR) were purchased from Sinopham Chemical Reagent Co. Ltd. Deionized water was used throughout.

***Bromination of poly (2,6-dimethyl-1,4-phenylene oxide) (BPPO)*:**

PPO was brominated via free radical bromination as previously reported^1^. A typical synthetic procedure is described as follows: To a stirred solution of PPO (6 g, 50 mmol) in chlorobenzene (60 mL) was added N-bromosuccinimide (NBS) (3.6 g, 20 mmol) and 2,2-azobis(2-methylpropionitrile) (0.3 g). The reaction mixture was heated at 140 ^o^C for 8 hours. Afterwards, it was poured into excess ethanol to form light brown precipitate of BPPO. Then, the polymer was redissolved in chloroform (60 mL) followed by precipitation in ethanol again. After dried at 60 ^o^C for 48 hours, BPPO was obtained as light brown fibers with a bromination degree of 0.23 (Characterized by NMR employing CDCl_3_ as solvent).

***Synthesis of 6-(dimethylamino)-N-ethyl-N,N-dimethylhexan-1-aminium bromide (DMAQA)^2^:***

DMAQA was synthesized as previously reported with minor modifications. As depicted in Figure S1, 2.1 mL bromoethane in 15 mL ethanol was added to a stirred solution of 6 mL TMHDA in 75 mL ethanol under nitrogen atmosphere. The reaction was carried out at room temperature for 48 hours. Ethanol was evaporated in vacuum and the residue was washed by ether for several times. The white precipitate was collected by filtration and then added to 50 mL of acetone. After filtration, acetone was removed in vacuum and the product was obtained as white hygroscopic powder. Pure DMAQA was obtain by recrystallization from acetone/ether mixture (Yield=37 %). NMR spectra of DMAQA were obtained employing D_2_O as solvent.

***Synthesis of 4-bromo-N,N,N-trimethylbutan-1-aminium bromide (BrQA)^3^:***

BrQA was synthesized as previously reported as depicted in Figure S1. To a stirred solution of 1,4-dibromobutane (10 mL) in THF (100 mL) was continuously bumbled dry trimethylamine gas at ambient temperature and pressure conditions for 3 hours. Afterwards, the reaction mixture was stirred at room temperature for 48 hours. The white precipitate was collected by filtration and washed with ether followed by dried in vacuum for 48 hours (Yield=89 %). NMR spectra of BrQA were obtained employing D_2_O as solvent.

Figure S1. Synthetic procedures of DMAQA and DMABQA.


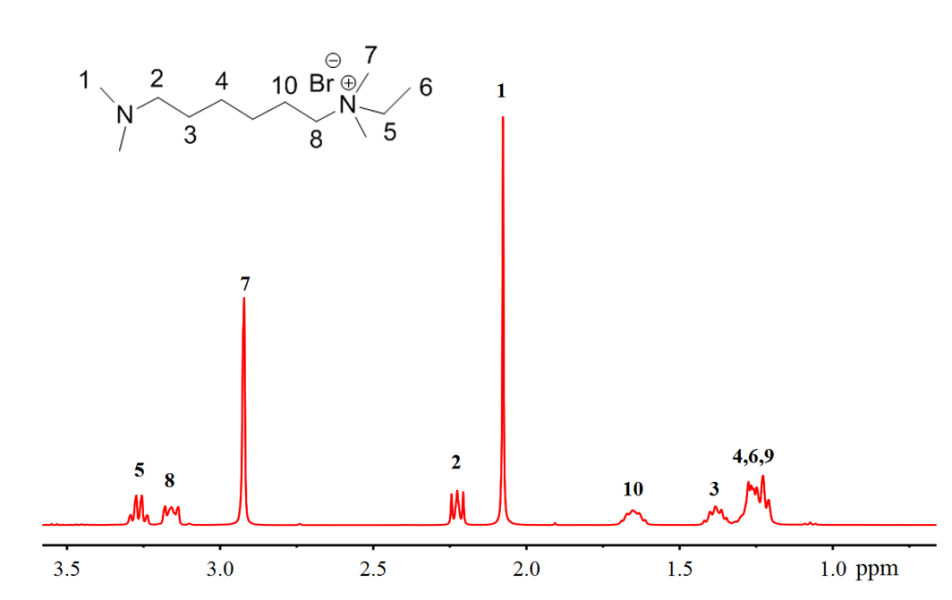


Figure S2. ^1^H NMR spectra of DMAQA.

***Synthesis of N^1^-(6-(dimethylamino)hexyl)-N^1^,N^1^,N^4^,N^4^,N^4^-pentamethylbutane-1,4-diaminium bromide (DMABQA)^4^:***

A solution of 2 g BrQA and 10 mL TMHDA in 30 mL acetonitrile was heated at 60 ^o^C for 24 hours. After filtration, the filtrate was concentrated in vacuum followed addition of excess ether. Afterward, the precipitate was collected by filtration and washed by ether to yield DMABQA as a white powder. It was purified by recrystallization and then dried at 60 ^o^C in vacuum (Yield=45 %). NMR spectra of DMABQA were obtained employing D_2_O as solvent.


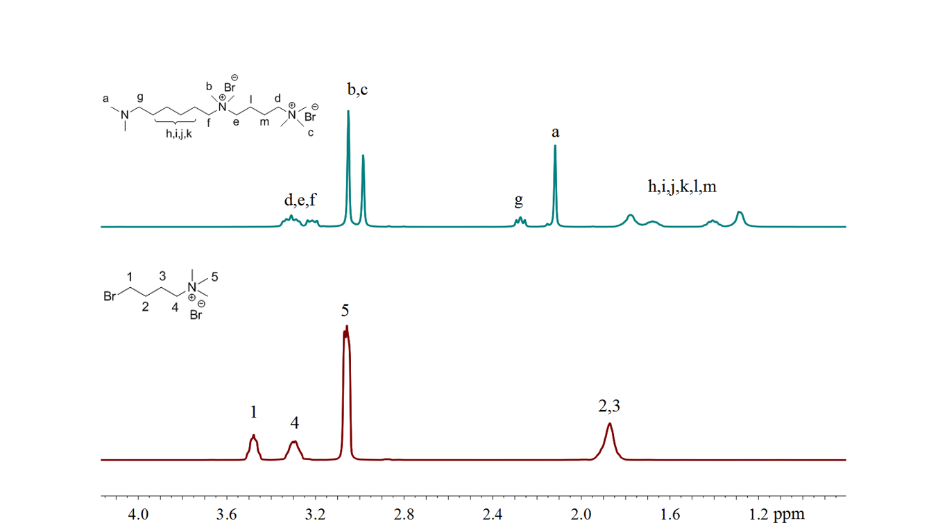


Figure S3. ^1^H NMR spectra of BrQA and DMABQA.

***Synthesis of multi-cations functionalized AEMs (BQAPPO and TQAPPO):***

To a stirred solution of 1 g BPPO in 10 mL NMP was added 1.2 equiv of DMAQA or DMABQA followed by stirring at room temperature for 24 hours. Afterwards, it was poured into excess ether and synthesized polyelectrolyte was collected by filtration and washed with ether for several times. After dried at 60 ^o^C for 24 hours, the polymer (1 g) was dissolve in NMP (15 mL) then casted onto glass plate and heated at 60 ^o^C to form transparent membrane. Key properties of the synthesized AEMs were listed in Table S1. NMR spectrums of BQAPPO and TQAPPO were obtained employing CD_3_OD as solvent.

Table S1. Key properties of synthesized AEMs.

| Membrane | Grafting ratio | IEC ^a)^ (mmol/g) | Water uptake ^b)^ (%) | Swelling ^c)^ (%) | | Conductivity ^d)^ (mS/cm) |
| --- | --- | --- | --- | --- | --- | --- |
| QPPO-0.25 | 0.25 | 1.47 | 48.7 | 9.1 | 12.9 | |
| QPPO-0.27 | 0.27 | 1.62 | 73.8 | 15.5 | 18.6 | |
| QPPO-0.33 | 0.33 | 1.88 | 99.5 | 21.1 | 29.5 | |
| QPPO-0.36 | 0.36 | 1.99 | 143.6 | 30.3 | 32.9 | |
| BQAPPO-0.10 | 0.10 | 1.22 | 8.9 | 3.2 | 16.5 | |
| BQAPPO-0.12 | 0.12 | 1.37 | 18.2 | 3.8 | 21.6 | |
| BQAPPO-0.15 | 0.15 | 1.56 | 42.5 | 11.1 | 36.3 | |
| BQAPPO-0.23 | 0.23 | 2.13 | 120.4 | 24.1 | 53 | |
| TQAPPO-0.10 | 0.10 | 1.72 | 55.6 | 9.2 | 43.3 | |
| TQAPPO-0.12 | 0.12 | 1.93 | 67.2 | 15.7 | 50.3 | |
| TQAPPO-0.15 | 0.15 | 2.07 | 76.2 | 16.6 | 57.9 | |
| TQAPPO-0.17 | 0.17 | 2.24 | 86.2 | 18.9 | 69.2 | |

a) Measured by Mohr method. b) Measured at 25 ^o^C, OH^-^ forms. c) In plane swelling ratio at 25 ^o^C in OH^-^ form. d) Measure at 25 ^o^C in fully hydrated conditions.

**Characterizations:**

^1^H NMR spectra was performed with an AV III 400 NMR spectrometer (^1^H resonance at 400 MHz, Bruker). Tapping mode atomic force microscopy (AFM) was recorded by a Veeco diinnova SPM, using micro-fabricated cantilevers with a force constant of approximately 20 N m^-1^. Mechanical properties of synthesized AEMs were measured by a DMA Q800 V20.24 Build 43.

***Water uptake, swelling ratio and ion exchange capacity***

A sample of synthesized AEM (1 cm in width and 4 cm in length) with a given mass was immersed in deionized water at room temperature for 24 hours. After that, the sample was taken out and excess water on the surface was removed by wiping the membrane with tissue paper. Length and mass of the hydrated membrane were measured quickly and liner expansion ratio (LER) was calculated as follows:

$LER=\frac{L_{w}-L_{d}}{L_{d}}\times100\%$ (1)

L_w_ and L_d_ were defined as the length of the sample in hydrated and dehydrated conditions separately. Similarly, water uptake (WU) of the membrane can be calculated by the following equation:

$WU=\frac{W_{w}-W_{d}}{W_{d}}\times100\%$ (2)

where W_w_ and W_d_ were defined as the mass of the sample in hydrated and dehydrated conditions separately.

Ion exchange capacity (IEC) of the prepared AEMs was measured by the conventional Mohr method. One sample of the membranes was firstly immersed in 1 mol/L NaCl aqueous solution at room temperature, then dried at 80 ^o^C in vacuum overnight. After the mass of the sample was recorded, it was immersed in 0.5 mol/L Na_2_SO_4_ aqueous solution for another 24 hours to release Cl^-^ from the membrane. Lastly, the solution was titrated with 0.1 mol/L AgNO_3_ aqueous solution employing K_2_CrO_4_ as indicator. IEC was calculated as follows:

$IEC(mmol/g)=\frac{V_{Ag^{+}}(mL)\times0.1(mol/L)}{W_{Cl^{-}}(g)}$ (3)


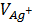
 was the amount of AgNO_3_ solution consumed while titration and
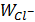
was the mass of the sample in Cl^-^ form.

***Hydroxide Conductivity***

Hydroxide conductivity of synthesized AEMs was measured by conventional four-point probe technique employing an Autolab PGSTAT 30 (Eco Chemie, Netherland) in galvanostatic mode and with an a.c. current amplitude of 0.1 mA and a frequency range of 1 MHz to 100 Hz. Bode plots were used to determine the frequency region over which the magnitude of the impedance was constant. Afterwards, the ionic resistance was obtained from the associated Nyquist plot. A sample of synthesized AEM was set into a Teflon cell and the membrane was in contact with 2 current collecting electrodes and 2 potential sensing electrodes (the distance between the potential sensing electrodes was 1 cm). Then, the cell was quickly immersed in deionized water in order to minimize the potential error caused by reaction of the hydroxide ions in the AEM with dissolved carbon dioxide. Afterwards, the impedance spectrum was collected. The ionic conductivity (κ) was calculated according to the following equation:

(4)

where L is the distance between potential sensing electrodes, R is the membrane resistance, d and W are the thickness and width (1 cm) of AEM sample respectively. The samples were equilibrated at the pre-determined temperatures for at least 30 min before measurement.

***Estimation of the alkaline stabilities***

A sample of synthesized AEM was soaked in aqueous 1 mol/L KOH solution at 60 ^o^C for increasing lengths of time. Afterwards, the membranes were immersed in distilled water and wash frequently for 48 h to remove the residual KOH. Afterwards, hydroxide conductivities and mechanical properties of the samples were again measured.

Table S2. Mechanical properties of QPPO, BQAPPO and TQAPPO AEMs (in OH^-^ form).

| Membrane | IEC (mmol/g) | TS (MPa) ^1)^ | E_b_ (%) ^1)^ | TS (MPa) | E_b_ (%) | |
| --- | --- | --- | --- | --- | --- | --- |
| QPPO-0.36 | 1.99 | 8.36 | 9.43 | brittle ^3)^ | brittle ^3)^ |  |
| BQAPPO-0.23 | 2.13 | 5.55 | 19.5 | 3.77 ^2)^ | 20.2 ^2)^ |  |
| TQAPPO-0.17 | 2.24 | 6.55 | 55.1 | 3.42 ^2)^ | 25.0 ^2)^ |  |

1) Measured before alkaline treatment. 2) Measured after soaking in 1 M KOH for 15 days.

3) Measured after soaking in 1 M KOH for 10 days.

**Reference**

1. Li N, Leng Y, Hickner MA, Wang CY. Highly stable, anion conductive, comb-shaped copolymers for alkaline fuel cells. *J Am Chem Soc* **135**, 10124-10133 (2013).

2. Li J*, et al.* Quaternary Ammonium Ionic Liquids as Bi-functional Catalysts for One-step Synthesis of Dimethyl Carbonate from Ethylene Oxide, Carbon Dioxide and Methanol. *Catal Lett* **141**, 339-346 (2010).

3. Bartsch RA, Zhao W, Zhang Z-Y. Facile Synthesis of (ω-Bromoalkyl)trimethylammonium Bromides. *Synth Commun* **29**, 2393-2398 (1999).

4. Hettich R, Schneider H-J. Cobalt(III) Polyamine Complexes as Catalysts for the Hydrolysis of Phosphate Esters and of DNA. A Measurable 10 Million-Fold Rate Increase. *J Am Chem Soc* **119**, 5638-5647 (1997).
